# Multikingdom microscale models

**DOI:** 10.1371/journal.ppat.1006424

**Published:** 2017-08-24

**Authors:** Layla J. Barkal, Erwin Berthier, Ashleigh B. Theberge, Nancy P. Keller, David J. Beebe

**Affiliations:** 1 Department of Biomedical Engineering, University of Wisconsin-Madison, Madison, Wisconsin, United States of America; 2 Carbone Cancer Center, University of Wisconsin-Madison, Madison, Wisconsin, United States of America; 3 Department of Chemistry, University of Washington, Seattle, Washington, United States of America; 4 Department of Urology, University of Washington School of Medicine, Seattle, Washington, United States of America; 5 Department of Medical Microbiology and Immunology, University of Wisconsin-Madison, Madison, Wisconsin, United States of America; 6 Department of Bacteriology, University of Wisconsin-Madison, Madison, Wisconsin, United States of America; Geisel School of Medicine at Dartmouth, UNITED STATES

## Introduction

**Fig 1:**
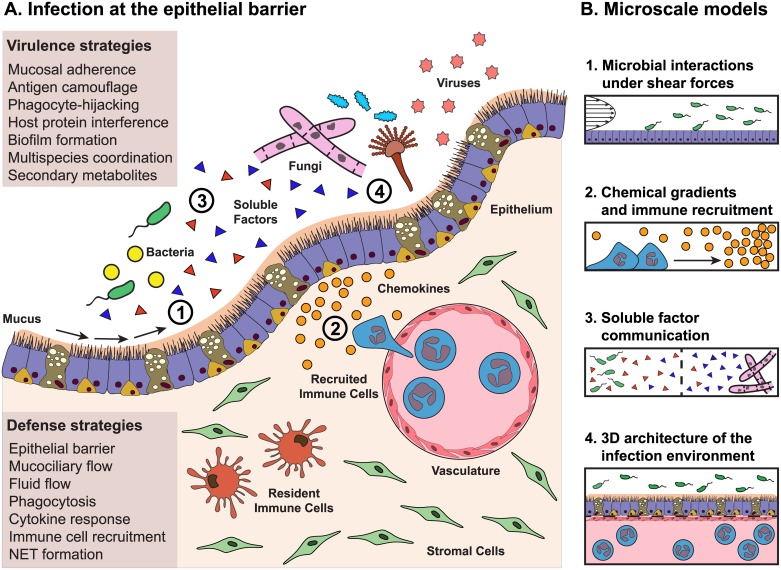
How microscale approaches can be applied to model the complex host pathogen microenvironment. (A) Virulence strategies of pathogens meet defense strategies of the host at the epithelial barrier. The infectious milieu can be incredibly complex, with organisms from different kingdoms interacting both with each other and with the host. Ideally, an accurate representation of the host, including the epithelium, vascular compartment, interstitial compartment, stromal cells, resident immune cells, and cytokines, would be exposed to an accurate representation of the invading pathogens, including a mixture of bacteria, fungi, viruses, and all sorts of soluble factors. In vitro methods now focus on making this complex environment experimentally tractable, modeling 1 or 2 components of the milieu. (B) Four aspects of the infection microenvironment that can be readily modeled using microfluidic approaches. 1. Microscale techniques simplify the process of modeling the fluid flows and shear forces that occur along the apical surface of the epithelium. 2. Gradients are easy to generate precisely and reproducibly in microscale and are ideal for performing chemotaxis assays. 3. Device geometry is customizable at microscale and offers control over coculture environments, allowing for soluble factor communication between 2 or more populations, as well as coculture between microbes from different kingdoms. 4. Microscale approaches can be used to create organotypic models that recapitulate aspects of tissue structure and function, which may better represent the host in host–pathogen interaction studies. Future microscale approaches may target entirely different aspects of the complex infection microenvironment depicted in (A).

Modeling infection in an immunocompetent host is complicated; complete models must factor in the various host defenses (e.g., cell barriers, mucociliary flow and other physical stresses present at the site of infection, recruitment of specialized immune cells) and virulence strategies of the pathogen (Fig 1A). These strategies include secretion of metabolites and proteins to derail the host immune response (for example, preventing neutrophil recruitment or degrading immune signals [1,2]), physically damaging host barriers (such as when fungal hyphae punch through fragile lung tissue [3]), and synergizing with other microbes of various kingdoms in our resident microbiomes (for example, when bacteria form biofilms only after sensing there are fungi nearby [4]) (Fig 1A).

In this Pearl, we discuss microscale systems—devices that operate with microliter volumes or that have geometries on the micrometer scale—as a versatile class of tools for studying these complex host–pathogen interactions. Microfluidic models of infection leverage the physics of the microscale to provide tunable shear forces (Fig 1B1) and chemical gradients (Fig 1B2) that are difficult to achieve at the macroscale. Microscale approaches enable spatial patterning of cell cultures and analysis of soluble factor communication (Fig 1B3), as well as construction of organotypic models that more closely approximate the in vivo architecture of the infection microenvironment (Fig 1B4).

## Microscale approaches to microbial interactions under shear forces

Fluids like mucus, blood, and urine play an active role in clearing the host of microbes by applying shear and binding forces that wash away pathogens and protect the epithelium from invasion (Fig 1A1). Physical forces encountered during an infection are typically a balance between clearing forces, such as shear forces applied to an organism by the movement of its environment, and adhesion forces through specific receptor–ligand interactions. Microscale systems are particularly well suited for models involving shear forces given their unrivaled control over fluid flow (Fig 1B1). For example, Anderson et al. use a microfluidic platform with continuous urine flow over a monolayer of human bladder epithelium to establish the first in vitro model of a complete cycle of uropathogenic *Escherichia coli* infection [5]. They show that the bacteria invade human bladder cells and replicate intracellularly, a known occurrence in urinary tract infections that is considered a possible source of recurrent infection [6]. It is only with continuous flow of urine that they observe the intracellular reservoir of bacteria begin to filament and eventually release, at which point the bacteria revert to rod forms that can infect neighboring cells, thereby completing the cascade of infection [5].

Shear forces can also be exploited by pathogens. Harker et al. describe a microscale model of *Toxoplasma gondii* tachyzoite infection where tachyzoites exhibit greater attachment to endothelial cells, with more gliding behavior and increased invasion and migration across the endothelial layer under flow conditions [7]. Weaver et al. describe how single *Staphylococcus epidermidis* bacteria bind fibronectin in low shear environments, while high shear conditions only permit binding of bacterial clusters [8]. This implies that forming clusters offers an advantage in conditions of higher shear. Shelby et al. present a model of the physical narrowing that is found in a capillary, which applies a shear force on erythrocytes. They observe that erythrocytes infected with *Plasmodium falciparum* lose functional deformability with shear in a manner that correlates with the parasitic life stage [9]. This matches clinical observations of severe malaria and offers a platform for understanding therapeutic interventions.

Although macroscale studies allow investigation of shear force in some situations [10], the microscale approach provides additional benefits. For instance, Harker et al. use microfluidics to apply a shear force while simultaneously observing single-cell behavior in that flow. Weaver et al. use laminar flow to pattern binding protein on channel surfaces and leverage a parallelized approach to compare 4 different shear stresses across the patterned surface, an experimental setup that would be challenging to achieve at macroscale. Finally, Shelby et al. use the flexibility of microfabrication techniques to recapitulate the geometry and scale of a capillary and its associated shear forces, an essential step in their study of how *P*. *falciparum* behaves in a capillary.

## Microscale approaches to chemical gradients and immune recruitment

Gradients of soluble factors play a crucial role in choreographing the immune response to infection (e.g., chemokine or microbial small molecule gradients) (Fig 1A2). Microfluidic systems can be designed in which diffusion forces dominate instead of convective mixing. As such, they are especially useful for generating and studying reproducible chemical gradients from basic 1D gradients (Fig 1B2) to complex 3D gradients [11,12].

Simple microfluidic chemotaxis assays are often used to study human immune cell migration in response to chemical signals that represent pathogen invasion. Berthier et al. describe a method that employs neutrophil migration as a way to screen fungal secondary metabolites for immunosuppressive function [13]. Using a microscale functional readout, they tested compounds that are difficult to purify in large volumes and reported that the metabolite endocrocin inhibits neutrophil migration, a finding validated in vivo in zebrafish. A similar approach was used to show that neutrophils from patients with sepsis migrate more slowly toward lipopolysaccharide, a component of the bacterial cell wall, than neutrophils from healthy patients [14]. In another study, *Aspergillus fumigatus* conidia were found to recruit neutrophils at a low level, but the addition of superimposed chemoattractant gradients both increased neutrophil recruitment and primed the neutrophils to kill conidia [15]. This suggests a role for the epithelial cells and other cells nearby an infection that are responsible for sounding the alarm; not only do their signals improve recruitment, they prime for a better functional response to the infection. Another integrated method was used to demonstrate that dendritic cells infected with *Salmonella typhimurium* respond differently to various chemokine gradients and, with a built-in positive-selection genetic screen, identified 7 *S*. *typhimurium* effector genes that contribute to this particular phenotype [16].

For chemotaxis assays, microscale approaches offer significant advantages including the ability to use less of difficult to purify chemoattractants as described by Berthier et al [13]. Additionally, laminar flow at microscale enables precise control over gradients, far beyond what is achievable at macroscale, where turbulent flow makes gradients inherently unpredictable at cellular scales. The precision and reproducibility of gradients was a key feature leveraged by each of the studies described above and contributed to the new biological insights. Microscale chemotaxis assays promise to enable yet further insights by allowing for individual cell tracking, which expands our ability to generate rich cellular migration datasets with metrics including how far individual cells migrate, their directionality, velocity, and cellular morphology.

## Microscale approaches to multikingdom coculture

The microenvironment of an infection often contains multiple microbial populations that can interact via soluble factor signaling as well as physically in specific spatial arrangements (Fig 1A3). Microbial cocultures are critical, as many small molecules of microbial origin are only expressed in mixed populations [17]. For example, *Cryptococcus neoformans* increases melanin (a known virulence factor) synthesis when in coculture with *Klebsiella aerogenes* [18]. Conversely, infection can cause local microbial populations to switch into pathogenic activity, as described with herpesvirus reactivation following infection with helminths [19]. Microscale platforms support microbial cocultures in large part by offering a level of control over the spatial distribution of cells and soluble signals (Fig 1B3), which facilitates coculture stability, avoiding overgrowth of any one species, and compartment-specific analyses.

Barkal et al. describe a platform designed to facilitate imaging and small molecule extraction and analysis of fungal and bacterial soluble factor cocultures [20]. They demonstrate clear morphologic changes in cocultures of *A*. *fumigatus* and *Pseudomonas aeruginosa*, 2 pathogens commonly found together in the lungs of patients with cystic fibrosis. Connell et al. present a 3D-printed platform in which a core microbial culture of *S*. *aureus* is encased by a shell of *P*. *aeruginosa*, which lends *S*. *aureus* resistance to beta-lactam antibiotics [21]. Kim et al. describe a triculture model in which human cells are patterned in between microbial islands, which have a layer of commensal organisms with enterohemorrhagic *E*. *coli* seeded on top. This setup allows for soluble factor communication with the host and direct communication between the 2 different microbial populations, pathogen and commensal [22]. The small molecule extraction platform from Barkal et al. improves our ability to access the soluble signaling molecules sent between microbial populations; the 3D nested shell structure from Connell et al. moves us closer to approximating the structure of in vivo granulomas, which are incredibly difficult to penetrate with antimicrobials; and the triculture model from Kim et al. allows for the study of how commensals protect against intestinal enterohemorrhagic *E*. *coli* infections.

## Microscale approaches to the 3D architecture of the infection microenvironment

Accurate modeling of infections will ultimately require reproduction of the subtle balance of the chemical milieu and the relative placement of all the specific cells and microbes in organs (Fig 1A4). It is clear too that mechanobiology, which considers the signaling effect of physical cues like stiffness and shear stress, matters for microbial behavior [23]. Technological innovations are occurring rapidly that allow microscale devices to incorporate microporous membranes, layered fluidics, and 3D-printed scaffolds to form specific spatial structures and to impart specific physical cues (Fig 1B4). Using these features, microsystems can be designed to mimic salient features of the in vivo organs, like the lung and the digestive tract. Kim et al. describe an approach to modeling the human gut using a single, differentiated human cell type, colonic epithelial cells, in a device that reproduces the apical and basal polarization of the epithelium. The polarization is achieved by applying a shear stress on the apical side of the culture and cyclical basal strains that mimics gut motility. This human gut model allows for the coculture of differentiated epithelial cells with strains of commensal bacteria [24]. A similar approach uses lumen structures made from 3D porous protein scaffolds lined with intestinal epithelium that also support bacterial coculture [25]. Other organotypic models of the human gut incorporate the capacity to coculture multiple species of gut microbes with human cells. Shah et al. describe a stackable model that enables coculture of both aerobes and anaerobes with differentiated host epithelial cells [26]. Costello et al. instead describe a method that recreates the villous structures formed by gut epithelium, which naturally creates niches preferentially occupied by different bacterial species. With this model, they show that commensal microbes protect against bacterial pathogens both by displacing pathogens and by preventing the adhesion of any newly introduced pathogens [27].

Organotypic models are also beginning to incorporate elements of the host immune system. Huh et al. describe a model of human alveoli with a flexible membrane lined on one side by alveolar epithelial cells and on the other by vascular endothelial cells. They demonstrate that *E*. *coli* added to the epithelial compartment can induce neutrophil migration from the endothelial compartment, across the membrane, and to the invading pathogens, which they subsequently phagocytose [28]. Microscale organotypic models are continually being extended to incorporate additional elements of the microenvironment and have already recapitulated bacterial invasion of gut epithelium and leukocyte recruitment through cellular layers to attack pathogens.

## Concluding remarks

The increases in physiological relevance enabled by microscale models present both opportunity and challenge. An interdisciplinary approach that includes the combined expertise of microbiologists, immunologists, and physicians is crucial for identifying which features of the infection context (Fig 1A1) are most critical to recapitulate, while discussions with engineers and biophysicists can determine how to combine these features into an effective model. Equally important is the development of strategies to best interpret these models. There is a unique opportunity for more sophisticated analysis than would be possible in traditional in vitro or animal models because microscale systems have tunable complexity and geometry. In that light, the input from geneticists, microscopists, analytical chemists, and systems biologists is critical to develop more targeted and relevant readouts from these advanced models. Methods to survey cocultures in real time, the ability to measure factor concentrations in 3D space, and methods to track metabolic changes in each community would be especially useful. Finally, given the scale of the parameter space that needs to be explored, biomathematicians and data scientists are key to building theoretical models of infection and to making relevant conclusions from the rich datasets being generated.

With these collaborations and cutting-edge technological innovations, microfluidic models of multikingdom interactions will offer biological relevance, versatility, and the ability to use small numbers of cells attainable from primary samples. These strengths make microfluidic models leading tools to study how microbial communities interact with human hosts during infection. Our understanding of pathogens and the broader context of infection promises to dramatically change how we understand human infectious disease and hopefully will give rise to novel therapeutic strategies to decrease the burden of these diseases.
